# Participants’ Perspectives on the iCareBreast Mobile-Based Perioperative Care Program for Women Undergoing Breast Cancer Surgery: Qualitative Process Evaluation

**DOI:** 10.2196/71686

**Published:** 2025-09-10

**Authors:** Yan Pang, Honggu He, Minna Pikkarainen, Swee-Ho Lim

**Affiliations:** 1 Alice Lee Centre for Nursing Studies Yong Loo Lin School of Medicine National University of Singapore Singapore Singapore; 2 National University Health System Singapore Singapore; 3 Digitalization of Healthcare Services Oslomet, Oslo Metropolitan University Oslo Norway; 4 Connected Health, Martti Ahtisaari Institute University of Oulu Oulu Finland; 5 KK Women's and Children's Hospital Singapore Singapore

**Keywords:** breast cancer, mobile health, psychosocial intervention, participant experience, qualitative study

## Abstract

**Background:**

Breast cancer treatment, particularly during the perioperative period, is often accompanied by significant psychological distress, including anxiety and uncertainty. Mobile health (mHealth) interventions have emerged as promising tools to provide timely psychosocial support through convenient, flexible, and personalized platforms. While research has explored the use of mHealth in breast cancer prevention, care management, and survivorship, few studies have examined patients’ experiences with mobile interventions during the perioperative phase of breast cancer treatment.

**Objective:**

This study aimed to explore the experiences of patients with breast cancer using iCareBreast, a mobile app designed to provide perioperative guidance and psychosocial support.

**Methods:**

A qualitative approach was used to explore participant experiences. A total of 13 English- or Chinese-speaking participants from the intervention group of a clinical study were recruited via purposive sampling between April 2021 and February 2022. Semistructured individual phone interviews were conducted, audio-recorded, and transcribed verbatim. Thematic analysis was performed to identify key patterns of experience, focusing on usability, emotional impact, perceived value, and areas for future improvement.

**Results:**

Overall, 4 main themes and 11 subthemes emerged from this study: (1) navigating the app with confidence and comfort, (2) making sense of treatment through relevant and evolving content, (3) finding emotional anchors in a time of uncertainty, and (4) advocating for broader use and continued motivation. Participants found the app user-friendly and appreciated its structure and locally relevant content, which helped reduce anxiety and enhance surgical preparedness. Features such as deep breathing exercises, motivational quotes, survivor stories, mindfulness practices, and peer support links offered emotional comfort and a sense of companionship. Participants strongly advocated for more personalized and adaptive content aligned with their treatment type and recovery progress. They also emphasized the value of interactive elements, such as video demonstrations and accessing messaging functions, to support sustained engagement. Many expressed the need for extended support throughout the adjuvant treatment phases, including chemotherapy and radiotherapy.

**Conclusions:**

The iCareBreast app was perceived as a supportive tool during the perioperative period, helping patients navigate both informational and emotional challenges. However, the findings underscore the importance of extending content across the treatment continuum and enhancing personalization and interactivity. mHealth interventions should be responsive to patients’ evolving needs and integrated into clinical care pathways to provide timely, comprehensive, tailored, and ongoing support for women with breast cancer.

## Introduction

### Background

Breast cancer is one of the most prevalent cancers among women worldwide and remains the leading cause of cancer diagnosis and mortality in Singapore [1,2]. Despite advancements in treatment, patients frequently experience heightened anxiety, depression, and uncertainty as they navigate surgical procedures and recovery [3-5]. Psychological distress affects approximately 50% of patients with breast cancer and is closely linked to factors such as short time since diagnosis, being in the preoperative phase, and concerns about body image [6]. Preoperative depressive symptoms are particularly concerning, with rates as high as 39% among patients undergoing mastectomy or lumpectomy, particularly in Asian countries [7]. These statistics highlight the significant psychological challenges faced by women during the perioperative period.

In addition to emotional distress, many patients report unmet information needs during the active treatment phase. During treatment preparation, patients often seek detailed explanations about the treatment plan and procedures, whereas their focus shifts to managing side effects and understanding long-term outcomes during treatment [8]. Supportive and goal-oriented strategies before surgery, including psychosocial interventions, have been shown to effectively reduce stress and improve the quality of life for patients with breast cancer [9-11]. These findings underscore the critical importance of comprehensive psychosocial support in addressing psychological distress and unmet information needs, ultimately improving outcomes during the perioperative period.

Traditional face-to-face psychosocial support mechanisms may not always be sufficient and convenient to meet the needs of patients with breast cancer, particularly during the brief and emotionally intense period from diagnosis to surgery. Mobile health (mHealth) interventions have emerged as innovative solutions that offer timely psychosocial support through accessible digital platforms. These interventions leverage flexibility, convenience, and cost-effectiveness to deliver personalized care. Research highlights mHealth’s value across breast cancer care, including prevention, early detection, care management, and survivorship. Through interactive features such as pictures, audios, videos, and tracking diaries, these platforms provide educational resources, monitor health, promote lifestyle changes, facilitate emotional support, and enable communication with health care professionals (HCPs) [12]. Despite their potential, psychosocial interventions specifically targeting patients with breast cancer during the perioperative period are limited, particularly those delivered through mHealth or eHealth platforms. Furthermore, there is a limited understanding of patients’ preferences for such solutions [13]. To address this gap, we developed an innovative e-supportive care mobile app, iCareBreast, designed for patients with breast cancer undergoing surgery. On the basis of the self-efficacy theory proposed by Bandura [14], the app aims to enhance patients’ self-efficacy in perioperative care while addressing their specific needs during this critical period.

### Objectives

Understanding participant experiences with these technologies is essential for uncovering the mechanisms behind their impact, optimizing their design to better meet patients’ needs, and guiding future implementation [13,15]. This process evaluation focused on exploring the experiences of patients with breast cancer who used the iCareBreast mobile app, designed to deliver care support during their surgical journey. By gathering qualitative insights into each component of the app, the evaluation aimed to identify user experience, focusing on usability, strengths, weaknesses, and suggestions for future improvements. These insights are critical for refining the app and ensuring that it effectively addresses patients’ needs, ultimately enhancing their perioperative care experience.

## Methods

### Study Design and Participants

This descriptive qualitative study is a follow-up to a randomized controlled trial (ClinicalTrials.gov ID NCT04172350) [16] that evaluated the effectiveness of iCareBreast, a mobile-based perioperative care program, on various health outcomes in improving perioperative self-efficacy, anxiety, depression, fatigue, health-related quality of life, and perioperative care satisfaction among women undergoing breast surgery in a public hospital in Singapore. The randomized controlled trial enrolled 123 women with early-stage breast cancer, randomized to either the iCareBreast intervention group (n=62) or the control group (n=61). While no statistically significant differences were found between the groups for most health outcomes, participants in the iCareBreast group reported higher perioperative care satisfaction.

All participants received standard multidisciplinary preoperative care, including consultations with a breast surgeon. The number and duration of consultations varied depending on case complexity and individual patient concerns. Patients were typically referred to a breast care nurse (BCN), who served as a nurse navigator providing education, emotional support, and care coordination. A short hospital stay (1-2 days) following breast-conserving surgery or mastectomy was typically required to ensure adequate pain control, postoperative monitoring, and wound care education. Postoperative follow-up appointments were scheduled with the breast surgeon. Medical social workers were available, and referrals were made based on clinical judgment and patient-specific needs. In addition to standard care, participants in the intervention group used the iCareBreast app daily at home.

### iCareBreast e-Support Care Program

The iCareBreast app was an interactive tool designed to guide and support patients through their perioperative journey. Tailored to each patient’s surgery date, the app provided daily guidance and information over a 29-day period (14 days before surgery, the day of surgery, and 14 days after surgery). Aiming to enhance care management and patient engagement for both surgery preparation and recovery, iCareBreast included 4 main functions: educational content, perioperative care guidance, psychological support, and social support.

The educational components covered presurgery and postsurgery topics relevant to breast cancer, such as anatomy, treatment options, and anesthesia. The perioperative care included preoperative preparation, day-of-surgery guidance, and postoperative care guidance on wound and drain management, pain control, infection monitoring, physiotherapy, and discharge planning. The psychosocial support component provided daily motivational quotes, mindfulness exercises, and stories from breast cancer survivors, along with a summary of breast cancer support networks available in Singapore. In addition, participants could ask questions through the app’s messaging system, with responses provided by HCPs and researchers within 24 hours.

The iCareBreast app was downloaded from the Google Play Store (for Android) or the Apple App Store (for iOS), available in participants’ preferred language—either English or Mandarin. A unique activation code was used for login, ensuring confidentiality and preventing cross-group contamination. Further details have been published elsewhere [16].

### Recruitment

Purposive sampling was used to select participants from the intervention group, categorized by age groups (21-49, 50-64, and ≥65 years), to ensure representation across different levels of technology competency. Participants in the intervention group were purposely invited to the interview phase at the same time they were enrolled in the main study. Interviews were conducted 2 weeks after surgery (immediately following the intervention) until data saturation was reached, which occurred with the 10th participant. Three additional patients with breast cancer were interviewed to confirm that no new information emerged, resulting in a total of 13 participants enrolled. All participants were informed that participation in the interview was optional and would not impact their involvement in the main study. They were also informed that the interviews would take approximately 10 to 20 minutes and would be audio-recorded.

### Data Collection

All semistructured interviews were conducted via phone calls during the COVID-19 period (from April 2021 to February 2022). Interviews lasted an average of 20 minutes, with duration ranging from 12 to 32 minutes. An interview guide was developed to facilitate discussions about participants’ experiences using the app, focusing on usability, strengths, weaknesses, and suggestions for future improvements.

Approximately 2 weeks after surgery, when participants had just completed their app use, a female research assistant who had no prior contact with participants trained in qualitative interviewing conducted individual phone interviews at mutually convenient times for both the interviewer and participants. The interview guide (Multimedia Appendix 1) was developed by an academic expert specializing in eHealth interventions, with another academic expert providing feedback on content clarity. A total of 10 interviews were conducted in English, and the remaining 3 in Mandarin. The Mandarin interviews were transcribed verbatim, then back translated into English by the same bilingual research assistant and verified for accuracy by another bilingual team member. Field notes were also taken to capture nonverbal cues (such as crying or laughing) to supplement the transcripts.

### Data Analysis

Data were analyzed following the 6 phases of thematic analysis described by Braun and Clarke [17]. This approach enabled us to explore and interpret patterns of meaning across the dataset, with an emphasis on understanding participants’ experiences and perspectives rather than counting specific responses. All interviews were transcribed verbatim by the same interviewer (research assistant) and cross-checked for accuracy by another team member. Data were analyzed concurrently with collection using inductive thematic analysis [17]. Two independent coders immersed themselves in the transcripts, repeatedly reading them. Codes were developed inductively from the data, and themes were shaped based on the depth and recurrence of meaning both within and across interviews. Initial coding was conducted independently by both coders line-by-line in the raw Microsoft Word documents, after which the codes were transferred to XMind Zen software to develop themes and subthemes related to participant experience [17,18]. A thematic map illustrating the connections between themes and subthemes was created after a third researcher reviewed and harmonized the findings [17]. Study rigor and reliability were maintained through prolonged engagement, peer debriefing, and by taking an audit trail and field notes [19]. Study finding was reported according to the COREQ (Consolidated Criteria for Reporting Qualitative Research) checklist (Multimedia Appendix 2) [20].

### Ethical Considerations

Ethics approval was obtained from the SingHealth Centralised Institutional Review Board (reference number 2019/2632). In accordance with the principles of the Helsinki Declaration, the participants were fully informed about the study, given ample time for consideration, and assured of their voluntary participation and right to withdraw at any time. All participants provided informed consent, and their identities were anonymized. They were then randomly assigned to either the intervention or control group. No compensation was provided to the participants.

## Results

### Participant Demographics

A total of 13 participants were interviewed for this study, all of whom were women with breast cancer undergoing breast surgery. The mean age was 58.5 (SD 11.8) years, with an age range of 35 to 73 years. Overall, 31% (4/13) of the participants were aged 21 to 49 years, 31% (4/13) were aged 50 to 64 years, and the remaining 38% (5/13) were aged ≥65 years. The average hospital stay was 2.85 days. Of the 13 participants, most were Chinese (n=11, 85%), with 1 (8%) Malay and 1 (8%) from another ethnicity. All participants identified as religious believers. Just more than half of the participants (11/13, 85%) received a polytechnic-level education or less; 46% (6/13) were currently employed, and 46% (6/13) had an income of more than SGD 3000 (approximately US $2243). Overall, 62% (8/13) of the participants underwent mastectomy, including 3 who received immediate reconstruction. Furthermore, 31% (4/13) of the participants underwent lumpectomy, with one receiving immediate postlumpectomy reconstruction. Approximately half of the participants (6/13, 46%) received adjuvant treatment (chemotherapy or radiotherapy) after surgery during the study period. Other sociodemographic and clinical characteristics of the participants are provided in Table 1.

**Table 1:** Participants’ sociodemographic and clinical characteristics (N=13).

Characteristics		Values
Age (y), mean (SD; range)		58.5 (11.8; 35-73)
**Age group (y), n (%)**		
	21-49	4 (31)
	50-64	4 (31)
	≥65	5 (38)
Hospital stay length (days), mean (range)		2.85 (1-9)^a^
**Marital status, n (%)**		
	Married	8 (62)
	Single	4 (31)
	Divorced or separated	1 (8)
**Ethnicity, n (%)**		
	Chinese	11 (85)
	Malay	1 (8)
	Others	1 (8)
**Religion, n (%)**		
	Buddhism	7 (54)
	Christianity	5 (38)
	Islam	1 (8)
**Education, n (%)**		
	No formal education	1 (8)
	Secondary school	7 (54)
	Polytechnic or college	3 (23)
	University	2 (15)
**Employment, n (%)**		
	Employed	6 (46)
	Unemployed	3 (23)
	Retired	3 (23)
	Others	1 (8)
**Monthly household income per capita (SGD $), n (%)**		
	≤1000	4 (31)
	1000-3000	3 (23)
	3000-5000	3 (23)
	>5000	3 (23)
**Type of surgery, n (%)**		
	Mastectomy	5 (38)
	Mastectomy with reconstruction	3 (23)
	Lumpectomy	4 (31)
	Lumpectomy with reconstruction	1 (8)
**Chemotherapy, n (%)**		
	Yes	6 (46)
	No	7 (54)
**Radiotherapy, n (%)**		
	Yes	5 (38)
	No	8 (62)

^a^Extended coordination of postdischarge care due to no immediate home support.

### Overview

**Figure 1:**
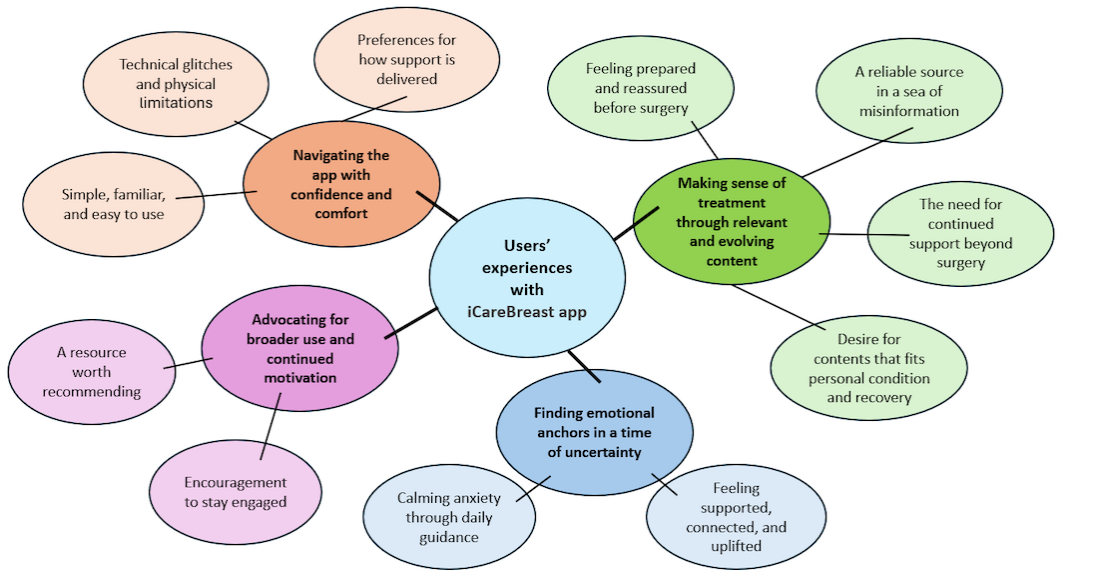
User experience thematic map for the iCareBreast app, showing 4 main themes and 11 subthemes.

Overall, 4 main themes and 11 subthemes emerged from the thematic analysis: navigating the app with confidence and comfort, making sense of treatment through relevant and evolving content, finding emotional anchors in a time of uncertainty, and advocating for broader use and continued motivation. A summary of the themes and subthemes is provided in Figure 1.

#### Theme 1: Navigating the App With Confidence and Comfort

##### Subtheme 1.1: Simple, Familiar, and Easy to Use

Participants generally found the app easy to use and navigate, with a clean layout that suited users across ages, including older adults. While the content was released daily, the app’s flexibility allowed them to preview upcoming content and catch up on any information from previous days that they may have missed. In addition, participants appreciated the daily app reminders, which helped engage them in completing their daily tasks consistently:

Simple, convenient. Also, there was no need to enter this, enter that, switch here and there.

P5

Yeah I appreciate the ah availability of the information before the actual day has passed.

P1

##### Subtheme 1.2: Technical Glitches and Physical Limitations

While most had a smooth experience of using the app, several users encountered login problems or faced issues such as the app hanging. However, these issues were quickly resolved by our technical support team in Finland, who developed the app and provided technical assistance throughout the study period. A few participants also noted that they were physically too unwell to use the app on the day of surgery and during the initial days after surgery, as they needed to remain in bed and focus on recovery:

That day there are some login technical issues. But then your colleague is setting it up. But then after that, this is no more issue.

P12

##### Subtheme 1.3: Preferences for How Support Is Delivered

Some participants missed out on using the in-app messaging feature due to its location or unfamiliarity, suggesting that making it more visually prominent would help them easily ask questions and receive support without needing to contact hospital staff. Others, particularly older users, preferred direct phone calls for urgent concerns. They noted that this feature is more appropriate for nonurgent issues. Many felt that shorter video-based instructions, particularly for physiotherapy or muscle relaxation exercises, would be easier to follow and maintain interest than written or static images:

I forgot to use, because it (messaging feature) was not part of the main interface, I think the design of that part was build it within the setting.

P4

I don’t know if it itself as a reminder to the patients. But my therapy is simple so I will remember after a few times you see, that I don’t need to see every, but if I keep seeing every day I will like I feel like boring...a video format will be good, because we can follow like an instructor that we can follow.

P12

#### Theme 2: Making Sense of Treatment Through Relevant and Evolving Content

##### Subtheme 2.1: Feeling Prepared and Reassured Before Surgery

Participants found the surgical guidance within the app clear and helpful, particularly as it walked newly diagnosed patients with breast cancer through the process step by step. Knowing what to expect for breast cancer and surgical procedures reduced uncertainty and helped them mentally prepared for surgery. The app’s detailed knowledge on postoperative expectations and postsurgery care such as pain management, wound care, and lymphedema prevention was particularly valuable:

I feel self-prepared (for surgery). Uh, I know what’s going to happen. what will happen. How am I going to accept it and take care of it.

P9

##### Subtheme 2.2: A Reliable Source in a Sea of Misinformation

Many participants felt that the content provided in the app was trustworthy and Singapore-relevant source of information. Many appreciated not having to seek additional resources, such as Google searches, which they found overwhelming or sometimes confusing. The volume and complexity of information shared during clinical visits often left them feeling overwhelmed and more likely to forget important details. Many participants appreciated that they could refer back to the app whenever needed, finding it a helpful resource to reinforce what they had learned during clinic visits:

Yeah, in fact this software is specifically for the patient at the hospital in Singapore, I think the content of the app is may more rigorous than my own online searching.

P1

So someone like me I need to refer to the app to refresh my memory. I’ll go back to the apps again and see the exercise (physiotherapy) and all those posted on the app lah.

P11

##### Subtheme 2.3: The Need for Continued Support Beyond Surgery

Several users felt the iCareBreast content ended too soon, particularly as new symptoms or emotional challenges often arose later. They expressed a strong desire to extend the app’s content to include the subsequent adjuvant treatment phase, with a particular emphasis on chemotherapy. They suggested adding information on different types of chemotherapy, side effect management, and ongoing recovery support to better prepare patients for this stage. Some participants also expressed interest in educational resources on radiotherapy, similar to the surgery-related guidance, covering topics such as types of radiation, key precautions, and postradiation care:

For me, the first 14 days were fine; my wound healed well, no pain, nothing unusual. But after the 14 days (end of app using), the pain started, and there was some discharge. Additional information could be helpful.

P7

Because like for me, the treatment after the surgery I think at least I have to go 9 more months of treatment so I think it would be helpful if we expand using the application after the surgery.

P8

##### Subtheme 2.4: Desire for Contents That Fits Personal Condition and Recovery

Some participants felt that, while the app provided general information, it lacked in-depth detail information based on their surgery type, treatment plan, or recovery stages. Participants felt that the app should adapt to their progress and treatment stages, with features such as adjusting physiotherapy exercises as they recover to provide more personalized support. They also hope for more guidance on nutrition, exercise, and long-term self-care as they progress in recovery:

Some people need chemotherapy and so on, so I think it would be great if it was added, it would be continuous for the patient. Because of this disease its treatment is relatively long…patient will just choose to see the content that relevant will be a long-term help for them.

P1

Additionally, this kind of information for faster recovery, like diet, I believe will be helpful, because I was trying to search if there’s any in the app, I couldn’t find much.

P3

#### Theme 3: Finding Emotional Anchors in a Time of Uncertainty

##### Subtheme 3.1: Calming Anxiety Through Daily Guidance

Many participants reported that the app’s day-to-day guidance and recovery tips gave them a clearer sense of what to expect, helping to reduce anxiety and alleviate doubts. The amount of daily information in the app also made them feel less overwhelmed. After practicing the provided deep breathing exercises or mindfulness practices, participants felt less pain and pressure, more relaxed, and experienced improved sleep quality:

The daily updates on what would happen each day, like meeting the anesthetist and what they would do. It helped me know what to expect, so I didn’t feel panicked.

P7

##### Subtheme 3.2: Feeling Supported, Connected, and Uplifted

The app served as a supportive emotional companion, helping participants feel cared for and less alone. They experienced a sense of connection, encouragement, hope, and optimism when reading stories from breast cancer survivors, realizing that many others had been through similar experiences. However, participants suggested adding more diverse breast cancer survivors’ stories to strengthen this emotional support aspect. They also appreciated the peer group resources provided, which allowed them to seek additional support and participate in more activities. In addition, participants reported feeling more focused, hopeful, and motivated to think positively and appreciate the present, inspired by the app’s motivational quotes and mindfulness practices. They suggested that adding more mindfulness practice videos would further enhance these benefits:

Yeah, I think it can introduce two people (breast cancer survivors’ story) a week, that make you feel there are a lot of people who are recovering... A kind of more encouragement.

P2

The app helped me a lot. One day, when I was feeling down, especially on Chinese New Year’s Day, I was very negative. Then, I saw content in the app that talked about people in Africa who don’t have food and children in other places who suffer from diseases. It reminded me to be grateful, and it completely changed my outlook. I stopped being resentful and started communicating better with my family.

P13

#### Theme 4: Advocating for Broader Use and Continued Motivation

##### Subtheme 4.1: A Resource Worth Recommending

Nearly all participants recommend the app to others in similar situations. They saw it as a valuable support tool that made a difference in both practical and emotional aspects of their recovery:

Yes, if others need something like this for the long term, I think I would recommend them to use.

P10

##### Subtheme 4.2: Encouragement to Stay Engaged

Participants expressed a desire for the app to provide ongoing motivational content to help them feel supported. Continued emotional support and inspiration were seen as key to encouraging long-term use of the app throughout their recovery:

We have this disease, diagnosis has been extremely scared, it is difficult to sleep. Have this thing (APP) is also a kind of encouragement, good. Isn’t it? If the app keeps giving me encouragement, confidence, of course I will continue using. Otherwise, if recurrence happen and the app content would be forgotten for a long time.

P6

## Discussion

### Principal Findings

This qualitative study explored how women with breast cancer experienced and engaged with a mobile perioperative support app during the breast cancer perioperative period. Through thematic analysis of interviews with 13 users, 4 key themes were identified, highlighting how the app supported users in navigating their surgery treatment journey, making sense of medical information, managing emotional challenges, and suggesting its potential beyond surgery.

The findings indicate that participants generally felt confident using the iCareBreast app due to its user-friendly interface and smooth navigation across both iOS and Android platforms. The app delivered daily content tailored to each user’s surgery date, which helped manage the information flow during an already emotionally overwhelming period. Participants valued this gradual delivery, noting it prevented the overload often experienced during clinic visits or conversations with others. The app’s flexibility was another key strength. Users appreciated being able to catch up on missed information if they enrolled late, and some users found it helpful to preview upcoming content if desired. Daily app reminders were seen as helpful prompts that supported consistent engagement, particularly by older users who also appreciated the app’s straightforward design. These features underscore the importance of designing digital tools that accommodate varying levels of digital literacy. A few users reported occasional app hang-ups or encountered login difficulties, particularly during scheduled app updates timed according to Finland’s time zone, where the iCareBreast app was developed and maintained, which inadvertently affected users in Singapore. These technical challenges were quickly addressed, ensuring minimal disruption to the participants.

Research consistently highlights that patients with breast cancer experience distress and anxiety during the perioperative phase due to uncertainty about what to expect, lack of health-related information, and concerns about unexpected surgical outcomes [21]. These emotional states can exacerbate feelings of being overwhelmed by the overwhelming volume of information provided during hospital visits [22,23]. Our study findings supported this observation. Many participants reported relying on the iCareBreast app to recall guidance from physicians or physiotherapists. This reliance underscores a communication gap that contributes to heightened distress early in the perioperative journey [21]. Although preoperative education and surgery-related information have been shown to reduce anxiety, improve knowledge, and enhance satisfaction, particularly when delivered in person [23], limited consultation time and the fast-paced nature of clinical care often restrict such opportunities. In this context, digital tools such as mobile apps can play a valuable role in supplementing patient education. Participants in our study noted that the iCareBreast mobile app helped in addressing their doubts. They appreciated its trustworthy, locally relevant content, which they described as more accessible and less overwhelming than online searches, which were often confusing or inconsistent. For many users, the app served as a reassuring and structured source of support during the perioperative phase.

Participants described the app as a source of emotional comfort and companionship during a time of uncertainty. Features such as deep breathing exercises, mindfulness practices, daily motivational quotes, breast cancer survivor stories, and links to peer support groups were seen as helpful in reducing feelings of isolation, enhancing focus, and increasing hope. These findings align with the self-efficacy theory proposed by Bandura [14] and the social support components, which guided the conceptual framework of the app’s content. The integration of practical knowledge with psychosocial support appeared to mitigate uncertainty-related anxiety and contributed to a more reassuring perioperative experience [21]. However, several participants expressed a desire for a greater variety of mindfulness videos and a wider selection of survivor stories. They felt that expanding these elements would offer additional encouragement, strengthen their sense of hope, and help them stay focused on the present. At the time of the study, the app included 3 survivor stories and 5 short mindfulness practices, which participants found limited. Enhancing this content may deepen the emotional support provided and increase the app’s impact on psychological well-being during the perioperative period [24].

At the same time, our findings point to areas for future development. Participants expressed a strong desire for the app’s content and motivational support to extend beyond the perioperative period, encompassing the adjuvant treatment phase, including chemotherapy and radiotherapy. They also highlighted the value of content addressing long-term needs, such as practical advice on nutrition and strategies for maintaining overall health. Many participants noted that their informational and emotional needs evolved significantly approximately 2 weeks after surgery, when discussions about further treatments typically began during follow-up consultations. These findings underscore the importance of providing continuous education and psychosocial support across the entire cancer treatment trajectory [25]. Expanding the app to support active treatment phases could enhance patient engagement, improve preparedness, and help reduce the anxiety associated with uncertainty. To achieve this, the content should align closely with the patient’s treatment progress and individual care plans. In addition, HCPs play a critical role in ensuring the ongoing and timely delivery of support that reflects each patient’s evolving needs.

In addition to extended support, participants expressed the need for more detailed and personalized information tailored to their specific type of surgery, treatment plans, and recovery process. They noted that personalized physiotherapy exercise and in-depth explanations of treatment options would enhance the app’s relevance to their individual experiences. This need for personalized information and care has also been emphasized in previous studies, which report that the complexity of breast cancer treatments, including various combinations of surgery, chemotherapy, radiotherapy, and hormonal therapies, creates a significant demand for individualized support [26]. To address these needs, future app iterations could incorporate features such as an initial intake assessment to identify individual treatment types, algorithms designed to tailor recommendations based on individual treatment plans, and interactive tools for progress monitoring [27-29]. These enhancements would enable the app to remain adaptable and relevant, providing patients with timely and personalized support as patients navigate the complexities of their breast cancer treatment.

In addition, participants expressed a preference for an interactive and engaging format. While the current app includes a messaging feature that allows participants to post nonurgent concerns related to breast cancer surgery, it was underused. Several participants mentioned that this feature was hidden within the settings, making them either unaware of its availability or prone to forgetting to use it. Others acknowledged its usefulness as an alternative means of communicating with HCPs but noted that its use was limited to nonurgent matters, and some preferred speaking with HCPs directly through a phone call. Importantly, many participants favored video content, particularly for deep breathing and physiotherapy exercises, because they found visual demonstrations easier to follow and more confidence building than text or image-based instructions. These findings highlight the need for mHealth interventions to prioritize interactive features to support long-term patient engagement. Future research delivering interventions via mHealth platforms should explore the integration of additional interactive components, such as enhanced video-based content, user-friendly messaging systems, and real-time feedback mechanisms. By aligning with patient preferences, such features have the potential to engage users more actively with digital health tools [30]. Aligning app design with patient preferences and use behaviors could increase engagement, enhance satisfaction with care, and contribute to improved health outcomes over time [13,27].

### Recommendations for Future Clinical Practices and Research

This study highlights the significant emotional burden faced by patients with breast cancer in the perioperative period. Many participants experienced heightened anxiety and distress due to the uncertainty of surgery and its potential outcomes. During this time, patients often felt overwhelmed by the volume and complexity of information provided by HCPs. The challenges are compounded by the short window between surgical decision-making and the procedure itself-often just a few days, depending on hospital scheduling and clinical urgency. This limited time frame can make it difficult for patients to process their diagnosis, seek clarification, or access psychosocial support, particularly as preoperative consultations are typically brief and infrequent. In light of these findings, HCPs, particularly BCNs, play a proactive role in regularly assessing and addressing patients’ informational, emotional, and psychosocial needs, particularly during the preoperative period. Early introduction of supportive resources, including digital tools, can help patients better prepare for surgery and reduce feelings of uncertainty. After surgery, as patients often transition quickly into adjuvant treatment, ongoing support remains crucial. BCNs play a vital role in maintaining ongoing communication and providing emotional support to help patients cope with the evolving demands of treatment and recovery.

Future research should focus on qualitative studies to explore patients’ experiences and support needs throughout the continuum of care, from the waiting period for diagnosis, the period of diagnosis to surgery, and the transition into postsurgical and adjuvant treatment. Such studies could provide valuable insights into how mHealth interventions can be optimized to benefit more patients by extending their use period and tailoring support to address patients’ evolving needs at each stage of their treatment journey. Further development of mHealth solutions should prioritize personalization, such as tailoring content based on individual treatment plans and recovery stages, to improve relevance and user engagement. Adding interactive elements such as real-time messaging and video demonstrations can make the app more engaging and easier to use. By designing features based on patients’ preferences, future versions of the app could play a stronger role in supporting better health outcomes and making oncological care feel more personalized and responsive.

### Study Strengths and Limitations

A key strength of this study was its use of mHealth technology to support patients with breast cancer during the perioperative period, a phase often marked by high anxiety and limited access to psychosocial support. The iCareBreast app provided timely, accessible, and structured guidance, which was particularly valuable during the COVID-19 pandemic when traditional face-to-face support was not feasible. This study is one of the few that specifically explores user experiences with mHealth, specifically during the perioperative phase of breast cancer care—a phase with limited research focus and unmet patient needs. It offers valuable practical recommendations for future psychosocial or educational interventions using mHealth and provides insights into enhancing patient engagement with mHealth solutions in clinical practice.

However, this study has several limitations. First, participants were restricted to those who were technologically proficient and willing to provide feedback, potentially limiting the generalizability of the findings to the broader population of patients with breast cancer. In addition, the perspectives primarily reflect those of older adults, with a mean participant age of approximately 58.5 years, which may not fully capture the age-specific perspectives of younger patients with breast cancer. Furthermore, as with other qualitative studies, reliance on self-reported data may introduce recall bias.

### Conclusions

In conclusion, this study provides valuable insights into participants’ experiences using a mobile perioperative support app and highlights the evolving nature of their informational, emotional, and psychosocial needs throughout the early treatment journey. Participants found the app to be a helpful and accessible resource that enhanced their preparedness for surgery, reduced anxiety, and offered emotional reassurance. However, their feedback also revealed a strong need for extended, personalized, and interactive support beyond the immediate postoperative period. These findings underscore the importance of designing patient-centered mHealth interventions that align with the dynamic realities of cancer care. Future iterations should prioritize content continuity, personalization, and engagement strategies to ensure long-term engagement across the treatment continuum. Such enhancements hold promise for improving patients’ experience, emotional well-being, and long-term outcomes in breast cancer care.

## Appendix

Multimedia Appendix 1 Interview guide for participants in the intervention group.

Multimedia Appendix 2 COREQ (Consolidated Criteria for Reporting Qualitative Research) checklist.

## Abbreviations

BCN: breast care nurse
COREQ: Consolidated Criteria for Reporting Qualitative Research
HCP: health care professional
mHealth: mobile health

## Data Availability

The datasets generated or analyzed during this study are available from the corresponding author upon reasonable request.
