# Supplementary material for: Participants’ Perspectives on the iCareBreast Mobile-Based Perioperative Care Program for Women Undergoing Breast Cancer Surgery: Qualitative Process Evaluation
Source: JMIR Hum Factors. 2025 Sep 10;12:e71686. doi: 10.2196/71686 (PMC12461162; doi:10.2196/71686)
Supplement: Multimedia Appendix 1 [file humanfactors_v12i1e71686_app1.docx]

**Multimedia Appendix 1: Interview guide for participants in the intervention group**

1. Describe your experience in using the iCareBreast mobile application in your perioperative care.
2. What are the strengths of the iCareBreast mobile application?
3. What are your thoughts and feelings about the iCareBreast mobile application in helping your knowledge about your surgical process?
4. What were the difficulties that you encountered while using the iCareBreast mobile application?
5. Would you recommend this iCareBreast application to other people who need it?
6. Do you feel the iCareBreast app useful? If yes, how? If not, why?
7. How do you feel that this iCareBreast app can answer any questions that you have without having to contact the hospital or care team member?
8. Which part of the iCareBreast mobile application is the most helpful for you?
9. Which part of the iCareBreast mobile application is the least helpful for you?
10. What are the suggestions you have to improve the iCareBreast mobile application?
11. Do you have any other comments about this iCareBreast app?
